# To end the neglect of neglected tropical diseases

**DOI:** 10.1016/j.lanwpc.2022.100388

**Published:** 2022-01-28

**Authors:** The Lancet Regional Health – Western Pacific

Neglected tropical diseases (NTDs) are a diverse group of tropical infections caused by various pathogens including viruses (eg, dengue or rabies), bacteria (eg, Buruli ulcer or yaws), protozoa (eg, human African trypanosomiasis), and parasitic worms or helminths (eg, dracunculiasis or soil-transmitted helminthiasis). Among the consequences of infections are pain, blindness, disability, disfiguration, and sometimes death. For example, Buruli ulcer is a debilitating skin and soft-tissue infection leading to permanent disfigurement and disability. Human African trypanosomiasis—with symptoms such as fever, lymphadenopathy, nocturnal sleeping pattern, and personality changes—is always fatal if untreated. Globally, WHO estimates that more than a billion people, approximately one in five individuals, are affected by NTDs. They contribute 19 million disability-adjusted life-years, which represents about 1% of the global burden of disease. Yet, as their name suggests, they have been historically neglected and often relinquished in the global health agenda. It was not until the landmark 2012 London Declaration on NTDs that they began gaining global recognition and attention. Since 2020, January 30 marks the annual World NTD Day, to raise awareness and engage the general public in the fight against NTDs. This year, we are embracing the third World NTD Day with the theme “Achieving health equity to end the neglect of poverty-related diseases”.

This theme reminds us of two intrinsic factors in NTDs: poverty and inequity. Due to the common geographical and social context of these pathogens (predominantly emerging in tropical areas with limited resources), NTDs mainly affect people living in the poorest and most marginalised communities. The discrepancy of disease burden between countries with different income levels in the Western Pacific region is a good example: the burden of 15 NTDs in 2019 was as low as 61·6 per 100 000 population in a high-income country such as Singapore, whereas this number markedly increased to 10·9 thousand per 100 000 population in a low-and-middle income country like China, and even higher in countries such as Vanuatu (35·2 thousand per 100 000 population) and Papua New Guinea (47·2 thousand per 100 000 population). NTDs are also characterised by inequity, which is closely associated with poverty, and in fact it is inequity that has given rise to neglect.

Inequalities are often multidimensional, and many interconnected layers can be recognised in NTDs. First and foremost, the disease prevalence among disadvantaged people across the globe with little public voice contributes to their neglect and inequality in receiving attention. Stigma due to cultural and social factors is a major obstacle to sufficient awareness, timely help-seeking behaviour, and proper treatment. The epidemiological setting—being endemic in low-income regions—often means limited economic incentives to fund research and treatments, leading to inequality in health services. Last but not least, NTDs are often overshadowed by other public health problems given their relatively lower mortality than other infectious diseases such as HIV/AIDS, tuberculosis, and malaria. This neglect has intensified during the COVID-19 pandemic, as financial and human resources have been further diverted.

Despite the relatively low mortality of NTDs, there will be tragic outcomes from continuing neglect: higher prevalence, worse health impact and morbidity, higher mortality in coinfections with other infectious diseases, stigma-related mental disorders, and exacerbated social inequalities. NTDs are, however, preventable and treatable. They can be prevented by improvements in clean water supply, sanitation, and hygiene. Mass drug administration (MDA) is an essential and efficient way to control NTDs. In this issue of *The Lancet Regional Health – Western Pacific,* a cluster randomised controlled trial found a video-based health education package significantly improved hygiene behaviour among Filipino schoolchildren and can be effective in preventing soil-transmitted helminths infections in schools in which the baseline prevalence did not exceed 15%. Another study in this issue assessing the safety of combined MDA for multiple NTDs in a population of more than 15 000 people in Papua New Guinea provides further support for the safety and feasibility of combined MDA and suggests new potential for the treatment of NTDs.

With global efforts, unprecedented progress has been achieved since 2012. 757 million people received NTD treatment in 2020 and 43 countries have eliminated at least one NTD. The first WHO roadmap on NTDs 2012–2020 has achieved some success, even with the impact of COVID-19. Dracunculiasis, one of the two NTDs that the roadmap aims to eradicate, is already on the verge of eradication, with only 54 human cases reported in 2019. Additionally, the reported number of new cases of human African trypanosomiasis, one of the four NTDs the roadmap aims to eliminate, have fallen by 98%, from 27 862 to 565 between 1999 and 2020. Other targets, however, will need further efforts. The Western Pacific is one of the two remaining WHO regions where Buruli ulcer is still prevalent. Numerous suspected cases of yaws are still reported in the region, with 13 694 cases in Solomon Islands in 2019 and notably 81 369 cases in Papua New Guinea in 2020. A cross-sectional survey in *The Lancet Regional Health – Western Pacific* also showed that the prevalence of impetigo observed in Samoan schoolchildren was one of the highest described globally. Altogether, these numbers suggest that the Western Pacific region should pay more attention to the continuing burden of NTDs in low-income settings, especially some of the Pacific Island countries.

Thoko Elphick-Pooley, the Executive Director of Uniting to Combat Neglected Tropical Diseases, said “As we have seen so far, this is an area in global health where we can achieve tremendous wins. Let us not miss the opportunity to end NTDs in our generation”. But first, let us try to end the neglect of NTDs, and increase efforts to contribute to the overarching 2030 global targets of the WHO roadmap 2021–2030 for the control of NTDs.

